# Words Matter: Content Analysis of Language Used When Documenting in the Medical Records of Patients in Mental Health

**DOI:** 10.2147/JMDH.S576861

**Published:** 2026-03-21

**Authors:** Monica Zolezzi, Shahd Abubaker Elamin, Oraib Abdallah, Yassin Eltorki, Turfa Alhathal, Esraa Maklad, Noriya Alkhuzaie

**Affiliations:** 1 College of Pharmacy, Qatar University, Doha, Qatar; 2 Mental Health Services, Hamad Medical Corporation, Doha, Qatar; 3 Aisha Bint Hamad Al-Attiyah Hospital, Hamad Medical Corporation, Doha, Qatar; 4 Pharmacy Clinical Services Unit, Sidra Medicine, Doha, Qatar

**Keywords:** stigmatizing language, recovery-oriented practice, clinical documentation, healthcare communication, person-centered care

## Abstract

**Background:**

Biased healthcare documentation adversely impacts patient outcomes, by affecting providers’ perceptions. Identifying stigmatizing language is crucial for appropriate workforce training.

**Purpose:**

To identify, describe, and compare language patterns used in medical records of individuals with mental illness.

**Methods:**

Patient encounters within the sole Qatari mental health governmental provider were randomly selected. Rich-text notes documented by any of eight eligible healthcare fields, written October–December/2021, and taking place during outpatient, inpatient, or community-homecare encounters were analyzed qualitatively for positive and negative language, and quantitatively for the percentage of notes with language types/subtypes. Association between language and patient/provider characteristics was tested using Goodness-of-fit chi2 and Fisher’s exact test, as appropriate.

**Results:**

Of 300 notes, most included potentially stigmatizing language (62.7%). Disability-first and inappropriate treatment-related language were the top isolated subtypes (33.22% and 31.31% of negative notes, respectively). Similarly, 66.67% of notes incorporated positive statements, mainly personalized language (44%). Positive language was statistically associated with specialty, age, diagnosis, discipline, setting, and patient’s first language. Stigmatizing language was statistically associated with setting, physician level, and discipline.

**Conclusion:**

Content analysis of mental health notes highlighted comparable use of positive and negative language. These findings urge policymakers and educators to advocate for recovery-oriented mental health documentation.

## Introduction

Stigma against mental illness has been deep-rooted for millennia across societies around the world, and it is often perpetuated by the words used to describe mental illnesses and those experiencing them.^1^,^2^ Language is used to communicate, educate and inform, but if not used appropriately, it can also confuse, mislead and alienate.^3^ In healthcare, language is one of the main practice tools used to transfer information between patients and healthcare providers (HCPs) and plays a major role in shaping therapeutic relationships.^2–4^

Even when records are only accessed by providers, using stigmatizing language in patient medical records can foster negative attitudes and biases among the providers themselves. Such language might shape the perceptions and decisions of providers through reinforced stereotypes, sometimes without even meeting the patient. Patients who are stigmatized may encounter a self-fulfilling prophecy, where negative impressions from past clinicians influence their interactions with new ones, ultimately leading to health disparities and marginalization within the healthcare system.^5^ In fact, growing research in this field highlighted that stigmatizing language in medical narratives, both spoken and written, can perpetuate ingrained biases.^6–8^ For example, in studies using case vignettes, clinicians who assessed the vignette with stigmatizing language describing patients were more likely to have negative attitudes toward them and provided an overall lower quality of care.^6–8^

Recently, studies have been conducted to identify and analyze stigmatizing language used by HCPs during documentation in patients’ medical records.^5^,^9^,^10^ In a qualitative study evaluating the written language used by physicians in ambulatory internal medicine, both negative and positive statements were documented in patients’ medical records.^5^ Some examples of the positive language identified were complementary language when describing the patient and minimizing blame. Conversely, negative language contained stereotyping terms, implying disbelief about patients’ reports/symptoms or questioning credibility.^5^ Similarly, >5% of clinical notes in obstetrics included terms classified as stigmatizing through questioning credibility, conveying disapproval, or stereotyping patients.^9^ In another recently published study;^10^ Out of 48,651 hospital admission notes, 1197 contained stigmatizing language most frequently about diabetes (599, 6.9%), substance use disorder (209, 3.4%), and pain (37, 0.7%).^10^

Nonetheless, although preliminary work on language as part of documenting care exists,^11^ important gaps remain. Studies were often limited to specific medical conditions (eg, diabetes^12^,^13^ or opioid use disorder^14^) documentation settings/timepoints (eg, admission or discharge notes^9^,^10^,^15^), or disciplines (eg, physicians,^5^,^16^ nurses,^16^ or pharmacists^17^), rather than examining language within general mental health services or across multiple disciplines and care settings (inpatient, outpatient, and community). This may limit insight into how language varies across settings and disciplines.

Akin to global contexts, evidence on the use of stigmatizing language in clinical documentation within Middle eastern and Arab healthcare settings remains limited, with stigmatizing language examined in the context of media portrayal.^18^,^19^ In the region, literature supports the presence and negative impact of stigma on patients with mental health conditions and the initiatives to combat it.^20–27^ In Qatar, for example, the Qatar National Mental Health Strategy (2013–2018), and the Qatar National Mental Health & Wellbeing Strategic Framework (2019–2022) are part of ongoing efforts to enhance mental health care.^27^,^28^ Given that approximately 1 in 5 individuals in Qatar experience mental health conditions^29–31^ and that documentation language may influence provider attitude and patient experience, examining positive and negative language in clinical documentation not only addresses local needs and practice but also contributes to the global understanding of language use in mental healthcare documentation.

To address these gaps, this study aimed to identify and describe the patterns of language used by different mental HCPs when documenting in their patients’ Electronic Health Records (EHR) across inpatient, outpatient, and community mental health services. The secondary objectives of this project included assessing the: 1) extent of use of positive and negative language subtypes among HCPs working in mental health, 2) relationship between mental health HCPs (documenters) and stigmatizing or positive language when documenting care, and 3) relationship between patient sociodemographic characteristics and stigmatizing or positive language.^5^

## Methods

### Study Design and Setting

This was a retrospective EHR chart review undertaken at Mental Health Services (MHS) at Hamad Medical Corporation (HMC), which is the main provider of secondary/tertiary healthcare in Qatar.

Qatar’s national healthcare system is predominantly financed by the government and serves both citizens and expatriates. The system is administered by the Primary Health Care Corporation (PHCC) and Hamad Medical Corporation (HMC), alongside private healthcare options, offering comprehensive services frequently free or subsidized. Established in 1979, HMC oversees over 14 facilities, and stands as Qatar’s principal healthcare provider for >2 million residents. The Mental Health Services (MHS) division of HMC, is the sole public provider of mental health services in the country. It offers a full care continuum across inpatient, outpatient, community, and virtual settings. MHS uses Cerner^®^.^32^ This electronic medical record system allows for the use of sensitive notes where access to notes is only limited to MHS staff across HMC.

Within the corporation, documentation practices may vary by service or discipline, but rely on Cerner Electronic Health Record [Cerner EHR] through Oracle Health.^32^ This system allows healthcare providers to access patients’ health records, communicate with other providers, and deliver/document healthcare clinical operations in an automated matter. In MHS, specifically, most of the documentation is done through rich-text/free-text notes with integrated forms. Although Arabic is the official language in the State of Qatar, English is widely spoken and is the language used in communications within governmental health care (including documentation in electronic medical records, regardless of the language of consultation or interaction with the patient).

### Sampling Procedure

Patient lists for those admitted to inpatient settings, attendees of outpatient clinics, and individuals who received home visits from the community team between October and December 2021 were sourced from the health information medical records departments. This three-month data collection period was selected to align with typical follow-up intervals in outpatient and community settings in the service, which average approximately three months. It also allowed for covering multiple inpatient admissions (average length of stay: 28 days) and several resident rotations (28-day blocks).

Subsequently, 300 unique patient records were randomly selected for review using a computer algorithm. This number was selected based on study objectives and previous literature to balance exploring meaningful patterns language use, adequate representation from multiple settings, and feasibility of coding and review by multiple researchers. From each of these 300 health record files, one random clinical note entry was chosen. The files were stratified to ensure the selection of 100 records from each of the three service settings: 1) inpatient care, 2) outpatient clinic appointments, and 3) community team home visits. Eligible notes could have been documented at any stage of the treatment process, including admission, follow-up, and discharge, and by any healthcare provider (eg, doctor/psychiatrist, pharmacist, nurse, psychologist, occupational therapist, physiotherapist, dietitian, social worker, case manager and students from all disciplines).

### Data Collection

From the patient’s records, the following data were collected: patient demographics (eg, age, gender, ethnicity, mental health diagnosis), characteristics of the HCP documenting care, and the type of encounter (inpatient, outpatient clinic or home care visit) All text within the selected chart notes was retrieved and entered into a Microsoft Excel^®^ spreadsheet along with patient, HCP/documenter, and encounter information.

### Coding Framework

A pre-defined coding framework of positive and negative language and subtypes (see Appendix 1) was developed to characterize the linguistic content of the encounter notes, derived from a comprehensive literature review of previous literature and organizational guidelines.^5^,^33–39^ Definitions and further information about each subtype are detailed in Appendix 1. For negative language, the subtypes were Disability Language/Disorder-first Language, Disapproval/Questioning Credibility, Stereotyping, in addition to Inappropriate treatment and medication-related Language. Conversely, for positive language, the subtypes were Person-first Language/Non-disabling Language, Personalized Language, Minimizing blame, and Bilateral decision making. For example, a free-text notes may include a statement with negative language such as “a schizophrenic patient” which would be coded as disorder-first language, as opposed to “a patient with schizophrenia” (coded as patient-first language). Using this framework, encounter notes were classified as “positive” (potentially conducive of recovery), “negative” (potentially stigmatizing), or both. The classification was based on the presence of at least one instance of positive/negative language as per the guide (Positive yes/no, Negative yes/no). Notes were allowed to be labelled as both positive and negative simultaneously.

To ensure the methodological rigour of coding, independent double-coding and iterative consensus were used. A training session for all research members was conducted to ensure coding framework understanding. Then, three researchers worked independently classifying the encounter notes, which were then reviewed against the coding framework by another three researchers for triangulation. The six research team members met regularly to discuss all coding and resolve any coding discrepancies.

### Data Analysis

Both qualitative and quantitative content analysis were used in this study. However, to maintain a clear distinction between linguistic choices on an individual level and predefined phrases used in departmental templates, phrases from standardized checklists/templates (both positive or negative) were excluded from the final quantitative and statistical analyses.

#### Qualitative Analysis

Deductive analysis was used to classify the extracted codes into subtypes of positive and negative language as per the coding framework. While free-text notes reflect documenters’ use of language, standardized templates may contain pre-set phrases that could unintentionally convey negative language (eg, smoker, compliant with her medications, dietary compliance). All coded statements from both free-text notes and standard forms were included in the qualitative analysis to capture all categories of positive or negative language present in the EHRs. Quotes were extracted from each category to illustrate the different types and subtypes of language.

#### Quantitative Analysis

Descriptive statistics (frequency and percentages) were used to report demographics and encounter characteristics. Then, using the categorized positive and negative statements, the number of notes in all subtypes/categories was quantified and tabulated against the sociodemographic characteristics of HCPs, patients, and settings where Pearson’s chi^2^ (goodness-of-fit chi2) and Fisher’s exact test were used as appropriate. Cramer’s V was calculated as a post-hoc test. Cohen’s omega was also used, and the effects were as follows: Small if w=0.10, medium if w=0.30, and large if w=0.50. Quantitative analyses were performed using STATA^®^ statistical software version 17 for Windows and Microsoft Excel.

## Results

### Demographics and Clinical Characteristics

A total of 300 encounter notes were analyzed both quantitatively and qualitatively. As displayed in Table 1, those notes were written by HCPs from eight different professions, with physicians and nurses having written 111 (37%) and 69 (23%), respectively. More than half of the notes (157, 52%) were written for patients following with adult psychiatry, and similarly in relation to patients identifying as Arabs (202, 67%). Most patients spoke Arabic (163, 54%) or English (117, 39%). Patients were mostly male (179, 59%), ages 26–35 years old (101, 34%), and diagnosed with schizophrenia/schizoaffective disorder (88, 29.33%), bipolar affective disorder (52, 17%), or major depressive disorder (44, 15%).

**Table 1:** Patient and Provider Characteristics (N=300)

**Patient Demographics and Clinical Characteristics**	**n (%)**
**Age Group**	
16–25	54 (18.00%)
26–35	101 (33.67%)
36–45	64 (21.33%)
46–55	40 (13.33%)
56–65	13 (4.33%)
≥66	28 (9.33%)
**Gender**	
Male	179 (59.7%)
Female	121 (40.3%)
**Ethnicity**	
Arab	202 (67.3%)
Southeast and West Asian (Chinese descent)	40 (13.3%)
South Asian (Indian subcontinent)	35 (11.7%)
White/Caucasian (European descent)	13 (4.3%)
Black (African heritage)	10 (3.3%)
**Mental Health Diagnosis**	
Schizophrenia and schizoaffective disorder	88 (29.33%)
Bipolar affective disorder	52 (17.33%)
Depression	44 (14.67%)
Others (Gender Identity, personality disorder, suicidal thoughts.)	32 (10.67%)
Learning Disability	26 (8.67%)
Anxiety disorders	23 (7.67%)
Alcohol/Substance use disorder	19 (6.33%)
Dementia	8 (2.67%)
Obsessive compulsive disorder	8 (2.67%)
**Primary Spoken Language**	
Arabic	163 (54.33%)
English	117 (39%)
Urdu	10 (3.33%)
Hindi	8 (2.67%)
Filipino	1 (0.33%)
Tamil	1 (0.33%)
**Documenter/Provider Characteristics**	**n (%)**
**Professional level for Physicians (n=111)**	
Resident	45 (15.00%)
Consultant/Sr. Consultant	41 (13.67%)
Specialist/Fellow	25 (8.33%)
**Total**	111 (37.00%)
**Discipline (N=300)**	
Physician	111 (37.00%)
Nurses	69 (23.00%)
Case manager	20 (6.67%)
Clinical Pharmacist	20 (6.67%)
Dietitian	20 (6.67%)
Occupational therapist	20 (6.67%)
Physiotherapist	20 (6.67%)
Social worker	20 (6.67%)
**Specialty (N=300)**	
Adult Psychiatry	157 (52.33%)
Community	49 (16.33%)
Geriatrics	30 (10.0%)
Learning Disability	27 (9.00%)
Forensic	26 (8.67%)
Women wellness	8 (2.67%)
Child and adolescents	3 (1.00%)

### Use of Negative and Positive Language

Upon analysis of the 300 encounters, positive and negative language belonging to all the pre-defined subtypes were extracted. Qualitatively, free-notes (HCP-authored) and structured sections (filled templates/checklists) in documentations from all disciplines revealed examples from both language types. Structured lists or templates included patterns such as the use of “smoker” or “obese” (negative, disease-first language), “diet compliance” (negative, inappropriate treatment-related language), or “Patient understands and agrees to plan” (positive language conveying bilateral decision-making). Table 2 presents a selection of the extracted quotes.

**Table 2:** Quote Examples of the Positive and Negative Language and Subtypes Extracted from the Clinical Notes

Category	Subcategory	Quote Examples
**Positive**	**Person-first Language/Non-disabling language**	“55 years old Qatari female with working diagnosis of depression with Anxiety and somatic symptoms”
	**Complimentary/Approving Language**	“The patient seems to be interested and promised to call the center tomorrow”
	**Personalized language**	“Feels overwhelmed with his challenges but hopeful of treatment with medical follow-up”.
	**Minimizing blame**	“The patient did not come to the appointment in person because she did not have a car”
	**Bilateral decision**	“Arranged also home visit on Thursday, 21/10/2021 and she accepted”.
**Negative**	**Disability Language/Disorder-first Language**	“Denied being mentally ill. known case of Bipolar I disorder since 2009, last relapse of mania was in 2019”
	**Disapproval/** **Questioning Credibility**	“Claimed husband brought her by force. Accused the husband of being oppressive and not supportive financially. MSE not done as patient refused to be seen”
	**Stereotyping**	“The patient is very difficult to convince about reducing his medications”
	**Inappropriate treatment and medication-related Language**	“Today she was prescribed aripiprazole which she refused to take at first… was maintained on Aripiprazole 15 mg and Escitalopram 10 mg with very poor compliance”

On the other hand, findings from quantitative content analysis uncovered 201 notes (67%) incorporating positive language. Notes with negative language were closely numbered, as 188 notes (63%) had examples of stigmatizing language. Notes with either language types were further categorized into priori subtypes. Table 3 illustrates these subtypes along with the counts and percentages of notes using them.

**Table 3:** Use of Negative and Positive Language

Language Type/Subtypes	n (%)
**Notes with Negative Language**	**188 (62.7%)**
Disability Language/Disorder-first Language	99 (33%)
Inappropriate medication-related Language	96 (32.00%)
Disapproval/Questioning Credibility	55 (18.30%)
Stereotyping	41 (13.70%)
**Notes with Positive Language**	**201 (67.00%)**
Personalized Language	131 (43.70%)
Person-first Language/Non-disabling Language	80 (26.70%)
Complimentary/Approving Language	75 (25.00%)
Bilateral decision making	71 (23.70%)
Minimizing blame	29 (9.70%)
**Total**	300 (100.00%)

### Language Used in Context of Specific Notes’ Characteristics

Several documenter and patient characteristics were associated in a statistically significant manner with language types. Psychiatry specialty, documenter discipline, encounter setting, age group, diagnosis, and patient’s main language of communication, were all found to be associated with the use of positive language. On the other hand, discipline, care setting, and physician level (in notes written by physicians specifically) were associated with the use of stigmatizing language. Table 4 summarizes the calculated p-values and Cramér’s V for all the variables.

**Table 4:** Inferential Analysis of the Use of Language in Context of the Different Variables

		Language					
		Positive Language			Negative Language		
		p-value	V	Effect	p-value	V	Effect
**Documenter-related variables**	**Specialty**	0.000*	0.33	**Medium to Large**	0.056	0.19	Small to Medium
	**Discipline**	0.000*	0.41	**Medium to Large**	0.000*	0.41	**Medium to Large**
	**Physician Level**	0.155	0.18	Small to Medium	0.037*	0.25	Small to Medium
**Patient-related variables**	**Setting**	0.000*	0.27	Small to Medium	0.011*	0.17	Small to Medium
	**Age Group**	0.010*	0.24	Small to Medium	0.082	0.21	Small to Medium
	**Ethnicity**	0.78	0.08	Small	0.944	0.05	Small
	**Gender**	0.185	0.11	Small to Medium	0.275	0.09	Small
	**Diagnosis**	0.000*	0.36	**Medium to Large**	0.783	0.15	Small to Medium
	**Spoken language**	0.045*	0.18	Small to Medium	0.636	0.11	Small to Medium

**Notes**: V, Cramer’s V; P-value, of fisher’s exact test; *, Statistically Significant; bolded text denotes significant p-value or medium to large effect.

Further analysis of the presence of positive and negative language across the presented documenter and patient attributes is presented below. Table 5 provides a structured, visual representation of how the patterns of positive and negative language manifest in the distinct categories within the different attributes. These patterns are presented with the overall percentage of positive and negative language (67% and 62.7% in 300 notes, respectively) as reference. For instance, as seen in Table 5, 26/30 (87%) of notes written by the geriatrics team had positive language, a high percentage of positive language compared to the overall percentage of the 300 notes. On the other hand, notes written by members in the child and adolescent psychiatry had 2/3 (67%) include positive language (close to the overall percentage), while those written by adult psychiatry team members had a lower-than-overall percentage of positive language with 95/157 (61.21%).

**Table 5:** Inferential Analysis of the Use of Language in Context of the Different Statistically or Non-Statistically Significant Variables

		Language											
		Positive Language						Negative Language					
		>72% (Preferred)		67.00%		<62%		>67.7%		62.70%		<57.7% (Preferred)	
				± 5%						± 5%			
**Documenter-related variables**	**Specialty**	Community	46/49 (93.88%)	Child and adolescent psychiatry	2/3 (66.67%)	Adult psychiatry	95/157 (61.21%)	NSS		NSS		NSS	
		Geriatrics	26/30 (86.67%)			Forensic psychiatry	14/26 (53.85%)						
		Women Wellness	6/8 (75.00%)			Learning disability	14/26 (53.85%)						
	**Discipline**	Nurses	51/69 (73.91%)	NSS		Dietitians	11/20 (55.00%)	Physicians	76/111 (68.47%)	Physio-therapists	12/20 (60.00%)	Case managers	10/20 (50.00%)
		Case managers	15/20 (75.00%)			Occupational therapists	11/20 (55.00%)	Nurses	54/69 (78.26%)			Clinical pharmacists	10/20 (50.00%)
		Physicians	92/111 (82.88%)			Physio-therapists	10/20 (50.00%)	Dietitians	18/20 (90%)			Occupational therapists	4/20 (20.00%)
						Clinical pharmacists	8/20 (40.00%)					Social workers	4/20 (20.00%)
						Social workers	3/20 (15.00%)						
	**Physician Level**	NSS		NSS		NSS		Residents	35/44 (79.55%)	NSS		Consultants	22/41 (53.66%)
								Specialists/Fellows	18/25 (72.00%)				
**Patient-related variables**	**Setting**	Outpatient	66/98 (76.35%)	NSS		Inpatient	89/153 (58.17%)	Outpatient	63/98 (64.3%)	NSS		Inpatient	86/153 (56.21%)
		Community	46/49 (93.88%)									Community	39/49 (79.59%)
	**Age Group**	46-55	32/40 (80.00%)	16–25	37/54 (68.52%)	26–35	55/101 (54.46%)	NSS		NSS		NSS	
		≥66	24/28 (85.71%)	26–45	45/64 (70.31%)	56–65	8/13 (61.53%)						
	**Ethnicity**	NSS		NSS		NSS		NSS		NSS		NSS	
	**Gender**	NSS		NSS		NSS		NSS		NSS		NSS	
	**Diagnosis**	Acute Psychotic Disorder	1/1 (100.00%)	NSS		Bipolar Disorder	32/52 (61.54%)	NSS		NSS		NSS	
		Major Depressive Disorder	41/44 (93.18%)			Substance Use Disorder	11/19 (57.90%)						
		Obsessive Compulsive Disorder	7/8 (87.50%)			Schizophrenia and Schizo-affective disorder	46/87 (52.87%)						
		Dementia	6/8 (75.00%)										
		Others ^a^	26/32 (81.25%)										
	**Language**	Neither Arabic nor English ^b^	15/20 (75.00%)	Arabic	117/163 (71.77%)	English	69/117 (58.97%)	NSS		NSS		NSS	

**Note**: All variables included in the table showed a statistical significance difference in the use of positive and/or negative language. Green text indicates a preferred direction of language use, defined as higher use of positive language and lower use for negative language, with >5% difference from the overall average. Red text indicated a non-preferred direction, defined as >5% lower use of positive language or higher use of negative language; Greyed out cells denote variables with a non-statistical significant relationship to the presence of positive or negative language; ^a^Others: Other diagnoses including Gender Identity, Personality disorder, suicidal thoughts, and others; ^b^Neither Arabic nor English: patients whose primary and only language in the electronic health records is listed as Filipino, Hindi, Tamil, and Urdu.

**Abbreviation**: NSS, Non-statistically significant.

## Discussion

This study investigates positive (recovery-oriented) and negative (stigmatizing) language in EHRs in the setting of mental health services in Qatar. To the best of our knowledge, it represents the first study in this part of the world, and in mental health settings.

The results of this study revealed that HCPs in the mental health setting utilize a similar extent of positive and negative language when documenting patient care. Although this was a promising finding, the extent of negative language is still significantly higher than what other similar studies have reported (62.7% in our study vs. 3–20% in other studies).^9^,^10^,^40–42^ Such differences can be partly explained by the many determinants in each setting, sample, and methodology of assessment. For instance, most available literature investigates stigmatizing language only from the perspective of physicians and nurses, and in specific settings or types of notes (eg, neonatal/birth notes and admission/discharge, respectively). Since the present study focused on the setting of mental health, a setting that requires a multidisciplinary approach in its nature, several professions and note types were encountered. Hence, it can be expected that further differences would be observed in language patterns used in records due to this diversity. In any case, the presence of negative language in close to 70% of the notes regardless of past literature calls for attention to the causes and solutions to such prevalence.

Another interesting finding is that documenter profession and setting (outpatient, inpatient, or community) were the variables statistically associated with the use of both negative and positive language. Scarce literature exists comparing the use of language in notes among different professions. However, this can theoretically be attributed to inherent differences in documentation practices between professions due to training and work culture. When analyzing notes linguistically through a graph matching algorithm, Boyd et al suggested that physicians and nurses document records differently not only in terms of concepts but also terminology.^43^ Moreover, although the practice of documentation ideally starts during college education, documentation can be affected by practice and training in service. It is highly possible that many HCPs would mimic the language used by their supervisors and colleagues in their work setting, consequently picking up stigmatizing language, or even copy/pasting previously written notes with negative language embedded in them.^44^,^45^ Ultimately, due to the multidisciplinary nature of mental healthcare, professionals’ roles do not exist in isolation. Notes from one profession may influence others both through picking up on their writing practices and shaping team perspectives and decisions. Importantly, the associations identified in this study are exploratory in nature and should be interpreted as indicative of potential patterns rather than definitive differences.

On a similar note, qualitative analysis revealed templates and/or checklists using negative language within notes from several professions. As such templates are often obligatory from the respective departments as part of documentation and do not represent the individuals but rather the department, negative/positive language detected in them was not included in the final quantitative analysis. Additionally, in the present setting of health services in Qatar, HCPs are consulted when creating electronic forms or checklists, but they are primarily written and designed by the medical records section. Research supports that although literature on structured forms and data entry proposes the inclusion of HCPs as advisory sources when creating these forms,^46^,^47^ this involvement is primarily through advising for clinical content, process integration, and user experience, rather than appropriates of language.

When looking at positive language, several variables affected its use. For example, data in this study suggest that notes written by providers in more specialized areas included more positive language. The percentage of positive language in notes written by HCPs from the geriatrics, women wellness, and community teams was higher than the overall percentage. This might have also contributed to notes about patients of older ages, including more use of positive language. Of note, although the language spoken by the patient showed statistical significance in association with positive notes, the p-value was barely significant (p-value=0.045), which was supported by the small-to-medium effect (Cramer’s V = 0.1833). Additionally, as notes about patients speaking a language other than Arabic or English did not exceed 20, it was difficult to draw meaningful conclusions. Further research is needed to explore the relationship between the use of positive/negative language and the language spoken by patients, especially in cases where language barriers may be present.

Interestingly, while six variables in total were statistically associated with the use of positive language in notes, only three were associated with negative language. This might suggest that the use of positive language may be more deliberate and hence associated with more documenter- or patient-related factors. On the other hand, the use of stigmatizing language was observed in a wide range of negative expressions and descriptors with few variables affecting its use. This suggests that many HCPs may unknowingly include negative language in their documentation regardless of patient characteristics. One potential factor leading to that could be variations in HCPs’ English language fluency. A person’s language proficiency may inadvertently contribute to selecting bias-perpetuating terms, serving as an example of implicit bias—a more subtle form of stigmatizing practice that has been demonstrated to negatively impact the level of patient care.^40^,^48^ However, it is essential to highlight that the extent to which language proficiency influences the use of stigmatizing language remains uncertain, and further investigation is needed to establish such a connection.

Lastly, it is crucial to acknowledge the complex and ever-changing nature of language. The meaning and connotations of language, and stigmatizing language by extension, can be influenced by several factors including time, place, and culture. Additionally, phrases or words may shift from positive to negative and vice versa.^49^ Such is the case with terminology like “addiction” and “hysteria”, which evolved from clinical terminologies to stigmatizing language, and were replaced by more neutral or accurate terms.^50^,^51^ More recently, guidelines on language surrounding weight and obesity in the clinical setting started calling for person-first language and replacement of charged adjectives like “morbid/extreme” obesity with neutral severity stages “I, II, and III”.^52^ Conversely, the use of person-first language with autism for instance has become controversial.^53–56^ Identity-first language (eg, autistic individual, neurodivergent) has gained endorsement from several members of the community as autism is regarded as an integral part of one’s identity and encouraged to be treated with positivity and pride^53^ This, of course, still varies by community. In the Arab and Middle eastern communities, autism-related identity-first language is still highly stigmatized.^19^ Further studies are needed to investigate the intricacy of how cultural differences might affect the use of language in documentation.

## Strength and Limitations

This study is not without limitations. For example, during analysis in context of characteristics, some subgroups had few notes. This might have been due to the short collection period or large number of characteristics. Nonetheless, although small numbers of notes might not have meaningful statistical significance, they could still have clinical significance and provide a baseline for possible future research. Additionally, as studies with large samples usually use computer science and language models, this study has the advantage of human analysis, providing opportunity for detecting stigmatizing and positive language through context rather than individual terms, keywords, or expressions. Additionally, as the collection of data was during the COVID-19 pandemic, this may have impacted generalizability due to potential disruptions in care delivery. Nevertheless, this period witnessed significant adaptations in mental health service delivery in Qatar. In response to the necessity for physical distancing, telepsychiatry was implemented through telephone/videoconferencing to ensure the continuity of outpatient psychiatric care, demonstrating a reduction in no-show rates.^57^ Consequently, rather than diminishing the study’s generalizability, the inclusion of data from this period represents an evolving model of care that may hold increasing relevance in both current and future psychiatric practice in Qatar and similar contexts. Lastly, existing literature and guidelines used for the guide were primarily designed by healthcare providers rather than patients. Patient and Public Involvement (PPI) could have offered valuable insight into additional types and/or examples of stigmatizing/positive language. Nonetheless, evidence on patient reactions to reading their medical records indeed supports several stigmatizing language categories identified in this study’s guide, including disease-first, credibility questioning or disapproval, and stereotyping language.^58^ Importantly, since the reviewed medical charts are primarily read by healthcare providers, the category guide appropriately fulfils the present study’s objectives.

In fact, many of this study’s strengths lie in its methodology. To achieve the study objectives, a meticulously created guide was used for data extraction and coding. Moreover, the research team used piloting, extensive training, guide edits, and regular meetings to ensure objectivity and consistency throughout the data collection and analysis process. Unlike most previous literature, which focused only on physicians and sometimes nurses, this study included notes from eight different professions in addition to students. It also analyzed different types of notes, rather than focusing on admission or discharge notes alone. To our knowledge, this is the first study to comprehensively investigate the patterns of both positive and negative language in the mental health setting and from a multidisciplinary lens. It is also the first to explore the relationship between language appropriateness and various documenter and patient characteristics.

## Implications

All in all, findings from this research highlight the intricate nature of language patterns in EHR, and the vitality of needed efforts on an individual, organizational, and societal level. Healthcare providers should seek awareness about stigmatizing language, recovery-oriented language, and their different subtypes. Providers should be educated not only to constantly self-assess their own documentation but also raise awareness when noticing negative patterns in their colleagues’. Training to recognize and use appropriate language should also be integrated in curriculums as part of university education and residency programs. Of course, it is difficult to promote the use of appropriate language within records in isolation of society. In fact, stigmatizing language is also prevalent in scientific evidence, guidelines, mainstream media, and day-to-day conversations. This paper, hence, calls for policymakers in healthcare and society alike to provide guidance on negative and positive language overall.

## Future Research

This research contributes to the body of literature related to stigma and stigmatizing language in the mental health setting and provides a baseline for a multitude of research opportunities. Future research can build and expand on this paper to evaluate language appropriateness in other settings, countries, or patient groups. Additionally, surveys or focus groups can be done with providers or patients to further explore individual characteristics affecting language appropriateness or appropriateness of language from other point of views. Engaging the public through Patient and Public Involvement (PPI) can present an essential opportunity to develop guidelines comprehensively reflecting perspectives of both healthcare providers and patients on the topic.

## Conclusion

This project investigated the language patterns used in the medical health records within the multidisciplinary mental health services in Qatar. Despite the high prevalence of positive language used by HCPs in the sample, the similarly predominant stigmatizing language use stresses on the vital need for radical changes in education, policies, and societal awareness. Further research exploring patient and provider characteristics in relation to language appropriateness is vital to create a future with recovery-oriented, stigma-free mental health electronic health records.

## Data Availability

Study data are available upon request from the corresponding author.

## Generative AI and AI-Assisted Technologies

During the preparation of this manuscript, the authors used artificial intelligence tools to assist with reviewing grammar and language of certain sections. The authors carefully revised and evaluated the content as necessary following this and assume complete responsibility for the published work.

## Abbreviations

EHR, Electronic Health Records; HCP, Healthcare Provider; HMC, Hamad Medical Corporation; IRB, Institutional Review Board; MHS, Mental Health Services; NSS, Non-statistically Significant; PHCC, Primary Health Care Corporation; PPI, Patient and Public Involvement; V, Cramer’s V.
